# DNA Encoding an HIV-1 Gag/Human Lysosome-Associated Membrane Protein-1 Chimera Elicits a Broad Cellular and Humoral Immune Response in Rhesus Macaques

**DOI:** 10.1371/journal.pone.0000135

**Published:** 2006-12-27

**Authors:** Priya Chikhlikar, Luciana Barros de Arruda, Milton Maciel, Peter Silvera, Mark G. Lewis, J. Thomas August, Ernesto T.A. Marques

**Affiliations:** 1 Department of Pharmacology and Molecular Sciences, The Johns Hopkins School of Medicine, Baltimore, Maryland, United States of America; 2 Instituto de Microbiologia Prof. Paulo de Góes, Universidade Federal do Rio de Janeiro, Rio de Janeiro, Brazil; 3 Southern Research Institute, Frederick, Maryland, United States of America; 4 Virology and Experimental Therapy Laboratory, Aggeu Magalhães Research Center, Oswaldo Cruz Foundation (FIOCRUZ), Recife, Brazil; 5 Department of Medicine, Division of Infectious Diseases, The Johns Hopkins School of Medicine, Baltimore, Maryland, United States of America; University of California, San Francisco, United States of America

## Abstract

Previous studies of HIV-1 p55Gag immunization of mice have demonstrated the usefulness of targeting antigens to the cellular compartment containing the major histocompatibility complex type II (MHC II) complex molecules by use of a DNA antigen formulation encoding Gag as a chimera with the mouse lysosome-associated membrane protein (mLAMP/*gag*). In the present study, we have analyzed the magnitude and breadth of Gag-specific T-lymphocyte and antibody responses elicited in Rhesus macaques after immunization with DNA encoding a human LAMP/*gag* (hLAMP/*gag*) chimera. ELISPOT analyses indicated that the average Gag-specific IFN-γ response elicited by the hLAMP/*gag* chimera was detectable after only two or three naked DNA immunizations in all five immunized macaques and reached an average of 1000 spot-forming cells (SFC)/10^6^ PBMCs. High IFN-γ ELISPOT responses were detected in CD8^+^-depleted cells, indicating that CD4^+^ T-cells play a major role in these responses. The T-cell responses of four of the macaques were also tested by use of ELISPOT to 12 overlapping 15-amino acids (aa) peptide pools containing ten peptides each, encompassing the complete Gag protein sequence. The two Mamu 08 immunized macaques responded to eight and twelve of the pools, the Mamu B01 to six, and the other macaque to five pools indicating that the hLAMP/*gag* DNA antigen formulation elicits a broad T-cell response against Gag. Additionally, there was a strong HIV-1-specific IgG response. The IgG antibody titers increased after each DNA injection, indicating a strong amnestic B-cell response, and were highly elevated in all the macaques after three immunizations. Moreover, the serum of each macaque recognized 13 of the 49 peptides of a 20-aa peptide library covering the complete Gag amino acid sequence. In addition, HIV-1-specific IgA antibodies were present in the plasma and external secretions, including nasal washes. These data support the findings of increased immunogenicity of genetic vaccines encoded as LAMP chimeras, including the response to DNA vaccines by non-human primates.

## Introduction

Several human immunodeficiency virus type 1 (HIV-1) antigenic formulations have been tested in animal models and clinical trials, but the immunogenicity and protection achieved to date are still far from the desired goals for an HIV-1 vaccine. While the correlates of immune protection are still only vaguely defined, it is now generally recognized that an effective HIV-1 vaccine must elicit strong T-cell responses as well as neutralizing antibody [1]–[4]. It has been postulated that the immunogenicity of a protein antigen can be enhanced by targeting the antigen to the major histocompatibility complex type II (MHC II) processing compartment of professional antigen-presenting cells (APCs). One of the approaches is the use of the lysosomal associated membrane protein–1 (LAMP) as a LAMP/antigen chimera to target endogenous antigens to the MHC II processing compartment [5]–[10]. Many laboratories have reported that LAMP targeting can greatly enhance the immune responses against a number of antigens, including HPV-16- E7 and E6 proteins; human telomerase reverse transcriptase (hTert); West Nile virus preM-E; the thyroid hormone receptor (TSHR); HIV-1 Gag, Env gp120, Env gp160, and Nef; human melanosomal antigen (MAGE-3); dengue 2 preM-E; listeriolysin O; and SARS coronavirus N protein [11]–[24].

Several approaches have been used to design DNA vaccines encoding LAMP/antigen chimeras. One frequently used construct contains the transmembrane and cytoplasmic (TMCy) domains of LAMP added to the C-terminus of the protein antigen. More recently, we have found that some antigens, including HIV-1 Gag, must be incorporated into the entire LAMP molecule in order to effect enhanced antigen expression and trafficking to the lysosomal compartment. Several LAMP/antigen chimeras, including proteins of West Nile, dengue, SARS CoV, HPV and HIV-1, have been shown to co-localize *in vitro* with MHC II, LAMP-1, LAMP-2, and H-2M in multiple cell types by confocal imunofluorescense microscopy, and/or by immunogold electron microscopy [11]–[16], [24].

The immunological benefits of LAMP-targeted antigens have been demonstrated in several mouse strains and, most importantly, also in humans [25]–[26]. LAMP/antigen chimeras have been shown to induce increased CD4^+^ responses to the antigens in several assay systems, producing increased secretion of IL-2, IL-4, IL-5 and IFN-γ cytokines; increased proliferative responses; a greater number of spot-forming cells (SFCs) in ELISPOT assays; intracellular cytokine staining (ICS); higher precursor frequencies; increased functional avidity and a broader response repertoire [11]–[24].

The increased CD4^+^-mediated responses produced by the LAMP/chimeras are thought to play an important role in modulating B-cell, CD8^+^ responses and the development of immune memory [11]–[24]. When compared to the non-targeted molecules, LAMP/antigens elicited greatly increased antibody titers, viral neutralization, antibody affinity and numbers of B-cell epitopes recognized. CD8^+^ responses were also enhanced in several LAMP/antigen chimeric systems, as assessed by tetramer staining, IFN-γ ELISPOT, chromium release, and the functional avidities and T-cell response repertoires of CD8^+^ cells. The longevity of the immunological memory of B cells and CD8^+^ cells is also increased in animals immunized with LAMP/chimeras.

Previous studies of HIV-1 DNA antigen formulations have described an HIV-1 p55gag DNA vaccine that elicited strong, broad and poly-functional cellular and humoral immune responses in immunized mice when the Gag sequence was incorporated into the complete LAMP cDNA sequence and the coding sequences were bracketed by the inverted terminal repeat (ITR) sequences of adeno-associated virus (AAV) [14]–[16]. These observations in mice led us to investigate the immunogenicity of a naked DNA encoding the HIV-1 LAMP/*gag* antigen formulation in Rhesus macaques, a relevant animal model for testing HIV vaccines for potential use in humans. We found that immunization of these primates with a human LAMP/*gag* (hLAMP/*gag*) DNA vaccine promoted a humoral and cellular immune response, which was associated with a sustained activation of B-lymphocytes as well as CD4^+^ and CD8^+^ T cells. Furthermore, Gag-specific T cells were detected after only two immunizations and were further expanded by booster injections of hLAMP/gag chimera. These results substantiate the usefulness of the LAMP vaccination strategy as a means of effectively eliciting T- and B-cell responses to naked DNA immunization of Rhesus macaques.

## Materials and Methods

### Plasmids

The mouse LAMP/*gag* (mLAMP/*gag*) plasmid was constructed as described previously [16]: Nucleotides 1–1503 of the HIV-1 HXB2 p55*gag* gene (GenBank^TM^ accession number K03455; HIV sequence database, Los Alamos National Laboratory Theoretical Biology and Biophysics, Los Alamos, NM, USA) was inserted into the pITR vector [27], which contains the AAV-ITR flanking the expression elements (cytomegalovirus promoter and bovine growth hormone polyadenylation signal). The p55*gag* sequence was inserted between the luminal domain and the TMCy domain of mouse LAMP (mLAMP) (GenBank^TM^ accession number J03881), as described previously [16]. In the present study, the complete mLAMP cDNA described above was replaced by the human LAMP-1 (hLAMP) cDNA (GenBank^TM^ accession number NM005561).

The control plasmids, consisting of mLAMP and hLAMP, were constructed using the respective complete LAMP sequences without Gag. The hLAMP/*gag* plasmid used for vaccination of Rhesus macaques was produced by Quality Biological Inc. (Gaithersburg, MD, USA), with a DNA purity of 96% and endotoxin level of 0.33 EU/mg.

### Analysis of protein expression

Human 293 cells were plated in 6-well plates (2×10^6^ cells/well) and transfected with plasmid DNA (4 µg) using the FuGENE^TM^ 6 (Roche Applied Science, Indianapolis, IN, USA) transfection reagents according to the manufacturer's instructions. The western blot analysis was done as previously described [15].

### Evaluation of hLAMP/*gag* targeting by confocal microscopy

MHC II (I-Ek)-expressing cells (DCEK.ICAM.Hi7, a gift of Dr. Susan Swain, The Trudeau Institute, Saranac Lake, NY, USA) [28] were plated onto poly-lysine-coated coverslips in 6-well plates (2×10^6^ cells/well) and incubated overnight. The cells were transfected with the DNA plasmids using Lipofectamine^TM^ 2000 transfection reagent (Invitrogen Life Technologies, Carlsbad, CA, USA), and 24–48 h later they were stained to evaluate cellular localization. Coverslips were fixed in 2% paraformaldehyde in phosphate-buffered saline (PBS) for 5 min and washed with PBS, then blocked and permeabilized with PBS containing 4% normal goat serum and 0.1% saponin. For detection of Gag expression, the cells were incubated for 1 h with mouse anti-Gag monoclonal antibody (provided by Dr. James Hildreth, Johns Hopkins School of Medicine, Baltimore, MD, USA) at a 1∶50 dilution. After three washes with 0.1% saponin in PBS, the cells were incubated for 1 h with Texas Red-labeled goat anti-mouse IgG (BD Pharmingen, San Diego, CA, USA) at a 1∶500 dilution. Co-localization of the hLAMP/*gag* chimera with MHC II of the transfected DCEK cells was performed by double immunostaining, first for the hLAMP/*gag* chimera proteins as described above and followed by anti-MHC II, by incubating the cells for 1 h with FITC-labeled goat antimouse I-Ek (14-4-4S) (BD Pharmingen) at a 1∶75 dilution. The cells were then washed three times with PBS, and the coverslips were mounted onto glass slides using ProLong Antifade reagent (Molecular Probes, Eugene, OR, USA). Confocal microscopy was performed using a Wallac confocal laser scanning microscope. Co-localization of the proteins with MHC II was thus determined by merging fluorophore images individually captured and digitally colored by use of Adobe Photoshop 7.0 (Adobe System Corp., San Jose, CA, USA).

### Studies with Mice

#### Immunization of mice

Female BALB/c mice, 6–8 weeks of age, were obtained from Charles River (Kingston, NY, USA). Mice in groups of eight were each immunized twice (21 days apart), intramuscularly (i.m.), with 50 µg of the indicated plasmid and sacrificed 7 days later. For CD8^+^ experiments, the mice were challenged 21 days after the first immunization with a VDK-1 vaccinia virus plasmid containing the native Gag sequence (10^7^ plaque-forming units; NIH AIDS Research and Reference Reagent Program, Rockville, MD, USA).

#### Antibody responses

Mouse serum was obtained from the tail vein 7 days after the second immunization. Serum IgG levels were measured by enzyme-linked immunosorbent assay (ELISA) as described previously [16].

#### Preparation of splenocytes for assaying the T-cell-mediated immune responses of immunized mice

Single-cell suspensions depleted of red blood cells were prepared from freshly isolated mouse splenocytes in culture medium (RPMI medium 1640 supplemented with 5% v/v FBS, 100 U/ml penicillin/streptomycin, 2 mM glutamine, 50 µM 2-mercaptoethanol, and 0.01 M HEPES buffer). Splenocytes were counted and resuspended at 10×10^6^ cells/ml in culture medium for T cell-mediated assays. T-cell responses of immunized mice were measured as described previously [15].

### Studies with Rhesus macaques

#### Rhesus macaques

Five healthy 4- to 8-kg male Rhesus macaques were maintained in the non-human primate facility of Southern Research Institute, Frederick, MD, USA. Animal care and treatment were in accordance with standards approved by the Institutional Animal Care and Use Committee, according to the principles set forth in the Guide for the Care and Use of Laboratory Animals, National Research Council, National Academy Press, 1996.

#### Immunization of macaques

Each animal was immunized intramuscularly (i.m.) five times at weeks 0, 4, 14, 22 and 38 with 5 mg of hLAMP/*gag* DNA plasmid using a biojector, and 18 blood samples were drawn over the 42 week experiment period. Mucosal samples including mouth swabs, nasal and rectal washes were collected after four DNA immunizations. Blood and mucosal samples of non-immunized macaques were supplied periodically as controls.

#### Isolation of peripheral blood mononuclear cells (PBMCs)

PBMCs were isolated by centrifugation (400×g, 40 min) on a Ficoll-Hypaque gradient (Amersham Biosciences Piscataway, NJ, USA). Mononuclear cells were collected from the interface and washed three times in cold phosphate-buffered saline with Ca_2_^+^ and Mg_2_^+^ (GIBCO, Grand Island, NY, USA). Residual red blood cells were removed with ACK lysing buffer (Quality Biological Inc., Gaithersburg, MD, USA). PBMCs to be used for cytokine analysis or ELISPOT assay were resuspended in supplemented culture medium (RPMI 1640 medium containing 1% FBS, 100 U/ml penicillin/streptomycin, and 2 mM L-glutamine).

#### CD8^+^ lymphocyte separation

Positive selection of cells expressing CD8^+^ antigen was performed with monoclonal anti-human CD8^+^ conjugated to R-phycoerythrin (PE) and microbeads conjugated to monoclonal anti-PE antibodies (CD8 MicroBead kit, Miltenyi Biotec, Bergisch Gladbach, Germany) as described by the manufacturer. The CD8^+^ cells were >98% pure as assessed by flow cytometry. Fractionated cells were suspended in RPMI-10 medium and used on the same day for ELISPOT assay.

#### HIV Gag-specific IFN-γ ELISPOT assay

The frequency of IFN-γ-producing CD4^+^ or CD8^+^ T cells from immunized Rhesus macaques was measured by ELISPOT assays, using the IFN-γ ELISPOT set from BD-Biosciences Pharmingen according to the manufacturer's protocol. Initially, ELISPOT plates were coated with anti-human IFN-γ Ig at 5 µg/ml and incubated at 4°C overnight. After blocking with RPMI-1640 containing 10% FBS for 2 h at room temperature (RT), total PBMCs (3×10^5^ cells/well) were cultured with hybridoma serum-free medium (GIBCO) supplemented with 1% FBS, 100 U/ml penicillin/streptomycin and 2 mM L- glutamine, in the presence of 10 µg/ml of HIVSF2 p55 gag recombinant protein (rGag); or HIV gag 15-amino acids (aa) peptides overlapping by 11-aa; or 20-aa overlapping by 10-aa. A negative control included in each assay consisted of medium lacking HIV antigen. Phytohemagglutinin (PHA) (Sigma, St. Louis, MO, USA) at 5 ng/ml with 500 ng/ml ionomycin (Sigma) was included as a positive control for each Rhesus macaque. After 16 h of culture, the plates were washed and incubated with biotinylated anti-human IFN-γ for 2 h at RT, followed by HRP-conjugated avidin for 1 h at RT. The reaction was developed with 3-amino-9-ethylcarbozole substrate (Calbiochem-Novabiochem Corporation, San Diego, CA, USA). Analysis of the IFN-γ levels was performed using an immunospot image analyzer (Cellular Technology Limited, Cleveland, OH, USA). Background spots obtained with medium alone were subtracted from each experimental value. All results were expressed as mean number of SFC per 10^6^ PBMCs. For each antigen, a responder Rhesus macaque was defined as one exhibiting a significant number IFN-γ SFC cells over the background value or value for non-immunized macaques, at any time point over the immunization course.

#### Analysis of IL-6 secretion by capture ELISA

Total PBMCs (5×10^5^ cells) from immunized macaques were cultured in a 96-well plate (Nunc, Roskilde, Denmark) containing recombinant human IL-2 (500 U/well). Except for the negative and positive control wells containing culture medium alone and 5 ng/ml PHA (Sigma) containing 500 ng/ml ionomycin, respectively, PBMCs were stimulated with 10 µg/ml HIVSF2 p55 rGag. After a 48-h incubation at 37°C in 5% CO_2_, the supernatants were collected for detection of secreted cytokines and stored at −20°C until further use. IL-6 concentrations were determined using a monkey IL-6 OptEIA^TM^ set (BD Pharmingen).

#### HIV Gag-specific humoral and mucosal immune responses

Serum samples were tested throughout the experimental period for the presence of binding antibody to HIV Gag. Nunc plates were coated with 5 µg/ml HIVIIIB lysate in sodium carbonate-bicarbonate buffer, pH 9.4 (Pierce, Rockford, IL, USA). After overnight incubation at 4°C, the solution was removed, and the plates were washed six times with PBS containing 0.05% Tween-20 (PBS-T) wash buffer. The plates were then incubated for 2 h at 37°C with blocking buffer (PBS-T with 5% FBS) and then washed three times with PBS-T. IgG responses were measured with four serial 1∶3 dilutions, starting at 1∶100, in blocking buffer. IgA responses in serum, nasal, mouth and rectal washes were measured with 100 µl of 10-fold diluted sample added to the blocked plate in duplicate. The plates were incubated overnight at 4°C. After six washes, 100 µl of horseradish peroxidase-conjugated rabbit anti-monkey IgG (Sigma) diluted 1∶5000 in blocking buffer was added to each well. For the detection of IgA antibodies, 100 µl of goat anti-monkey IgA (Nordic Immunology, Tilburg, Netherlands) diluted 1∶5000 in blocking buffer was added to each well. The plates were incubated for 2 h at 37°C and washed eight times with washing buffer. Turbo TMB substrate solution (BD Pharmingen) was then added to each well and incubated for 15 min at RT. The reaction was stopped by adding 100 µl of 1 M sulfuric acid, and absorbance at 450 nm was measured in a BIO-RAD model 3550 microplate reader.

#### B-cell epitope mapping

HIV-1 Gag 20-aa peptides, spanning residues 1 to 490 with a 10-aa overlap (NIH AIDS Research and Reference Reagent Program), were diluted in 0.1 M sodium carbonate-bicarbonate buffer, pH 9.4, to give a concentration of 5 µg/ml, and 50 µl of the individual peptide was added to each well of a 96-well plate, in duplicate. After overnight incubation at 4°C, the solution was removed, and the plates were washed six times with PBS-T. They were then incubated with blocking buffer for 2 h at 37°C and washed three times. Serum samples (100 µl of a 1∶300 dilution in blocking buffer) were added to each well, and the plates were incubated overnight at 4°C. After the plates were washed six times, 100 µl of HRP-conjugated rabbit anti-monkey IgG (Sigma), diluted 1∶5000 in blocking buffer, was added to each well. The plates were incubated for 2 h at 37°C and then washed. Turbo TMB substrate solution (BD Pharmingen) was added to each well and incubated for 15 min at RT. The reaction was stopped by adding 100 µl of 1 M sulfuric acid, and absorbance at 450 nm was measured in a BIO-RAD model 3550 microplate reader.

#### Statistical Analyses

All the graphs were made using GraphPad Prism Version 4.0a for Macintosh (GraphPad software, San Diego, CA, USA).

## Results

### Comparison of hLAMP/*gag* and mLAMP/*gag:* Gag Expression and Cellular Trafficking in Transfected Cells

**Figure 1:**
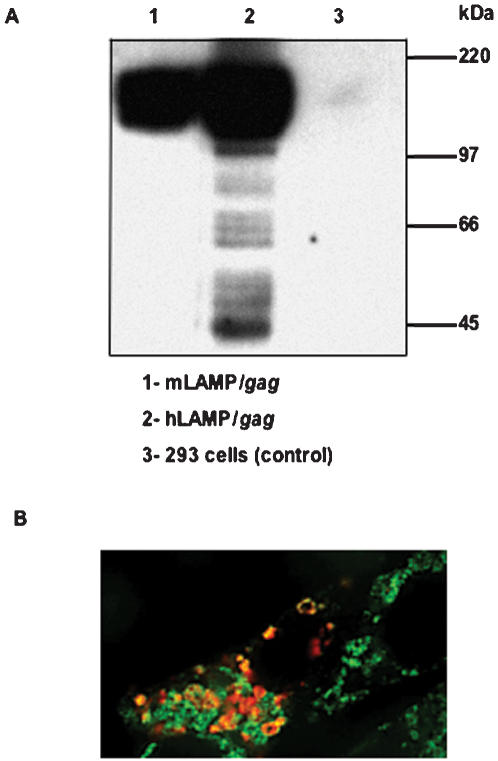
Expression and trafficking of mouse and human LAMP/*gag.* (A) Western blot analysis of human 293 cells transfected with mLAMP/*gag* and hLAMP/*gag* plasmids. Samples were probed with an anti-Gag monoclonal antibody. The molecular weight markers are indicated on the right. (B) DCEK cells were transfected with the hLAMP/*gag* plasmid and stained with anti-Gag (red) or anti-MHC II (green) monoclonal antibodies. Digitally merged image shows co-localization of the hLAMP/*gag* chimera- and MHC II-containing compartments (yellow).

LAMP/Gag expression and cellular trafficking to the cellular MHC II compartment has been repeatedly demonstrated in studies with murine LAMP chimeras [11]–[16]. The present study confirmed the similar expression and trafficking of the human LAMP/Gag protein chimera. Western blotting with anti-Gag antibody showed the presence of ∼200 kDa LAMP/Gag protein at comparable levels in 293 cells transfected with either mouse or human LAMP/*gag* (Figure 1A). There appeared to be increased degradation of Gag in the hLAMP/*gag* transfected cells; however, the significance of this is not known as the results of other similar studies of mLAMP/*gag* transfected cells have shown variable levels of Gag degradation products [14]–[16]. Co-localization of the hLAMP/*gag* chimera transgene product with the cellular MHC II was confirmed with transfected DCEK cells stained with anti-Gag and anti-MHC II monoclonal antibodies (Figure 1B).

### Immune Responses of Mice to hLAMP/*gag*

**Figure 2:**
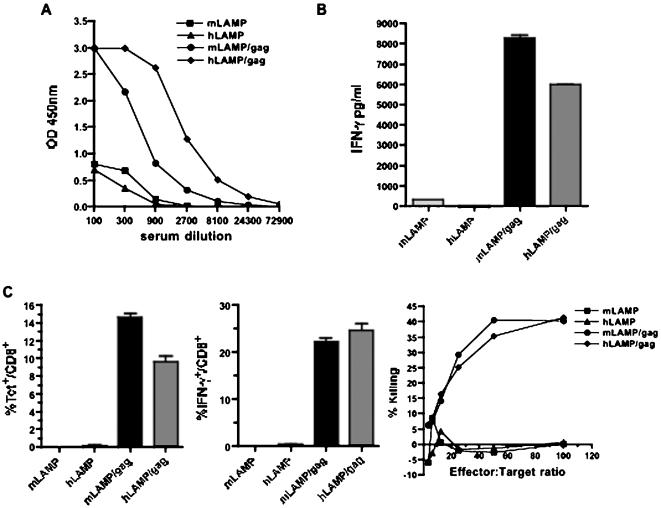
Immune responses in mice. Mice were injected i.m. on days 1 and 21 with 50 µg of the indicated pITR plasmids encoding mouse or human LAMP or LAMP/*gag* chimeras. (A) Humoral responses. Blood samples were collected 7 days after the second immunization. Each titration curve indicates the OD_450_ of Gag-specific total IgG present in serial dilutions of pooled serum from individual groups of mice. (B) CD4^+^ immune responses. CD4^+^ IFN-γ^+^ secretion from splenocytes of mice injected twice with the indicated plasmids. Animals were sacrificed on day 28, and splenocytes were prepared for assay of CD4^+^ T-cell responses and incubated in the presence or absence of antigen. A representative experiment is shown. Data are Mean±SD after subtraction of the background control values. (C) CD8^+^ immune responses. On day 21, mice were injected i.p. with 10^7^ PFU of rVV-*gag-pol*. Five days later, 10 µg of the H-2Kd-binding HIV-1 Gag peptide was injected i.v., and splenocytes were harvested after 2 h and analyzed *ex vivo*. Gag-specific CD8^+^ T-lymphocytes producing IFN-γ was quantified by flow cytometry and tetramer binding in the CD8^+^ splenic population. Cytolytic activity was also analyzed in a 4-h ^51^Cr release assay using P815 target cells pulsed with the H-2Kd-binding HIV-1 Gag peptide. The effector cells from the various groups of immunized mice were used as indicated. Nonspecific lysis (using unpulsed P815 target cells) was <5% for all groups (not shown). A representative experiment is shown.

Verification of the *in vivo* immune responses to the hLAMP/*gag* chimera and a comparison of the responses to mLAMP/*gag* were examined with mice immunized on days 1 and 21 with 50 µg DNA of the pITR plasmid vectors encoding the mLAMP/*gag* and hLAMP/*gag* chimeras. Total anti-Gag IgG responses were assayed with blood collected on day 28 (Figure 2A). The response of mice immunized with the hLAMP/*gag* construct was considerably greater than the response to the mLAMP/*gag*.

Splenocytes of mice sacrificed after the two immunizations were assayed for T-cell IFN-γ responses. The MHC II-targeted mouse and human LAMP/*gag* plasmids elicited comparable IFN-γ^+^ responses (8000 pg/ml and 6000 pg/ml, respectively) (Figure 2B).

Assays of CD8^+^ T-cell responses were carried out with mice immunized once with 50 µg of the plasmid DNA, followed 21 days later by *in vivo* expansion of Gag-specific T cells through inoculation with recombinant vaccinia-Gag-Pol (rVVGag-Pol). Five days later, the mice were injected with an immunodominant H-2Kd-restricted Gag peptide epitope and sacrificed after 2 h for the *ex vivo* assay. Mice immunized with both the mouse and human LAMP/*gag* chimeras uniformly developed significantly strong CD8^+^ responses as measured by epitope-specific CD8^+^ tetramer binding, intracellular IFN-γ staining, and CTL lysis of peptide-pulsed target cells (Figure 2C). The larger fraction of CD8^+^ T cells expressing IFN-γ (>20%) following inoculation with recombinant vaccinia virus encoding Gag, in comparison with the proportion of tetramer positive cells, is attributed to the massive *in vivo* expansion of Gag-activated CD8^+ ^T cells reactive against several other Gag MHC I epitopes, besides the immunodominant H-2Kd-AMQMLKETI peptide-tetramer epitope complex.

### Immunization of Rhesus Macaques with the hLAMP/*gag* DNA Plasmid

**Figure 3:**
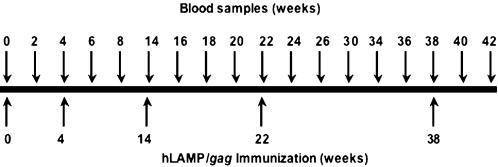
Protocol for immunization of Rhesus macaques with the hLAMP/*gag* plasmid. Immunization and blood sample collection from five Rhesus macaques immunized five times with 5 mg of hLAMP/*gag* DNA plasmid were carried out as shown.

**Figure 4:**
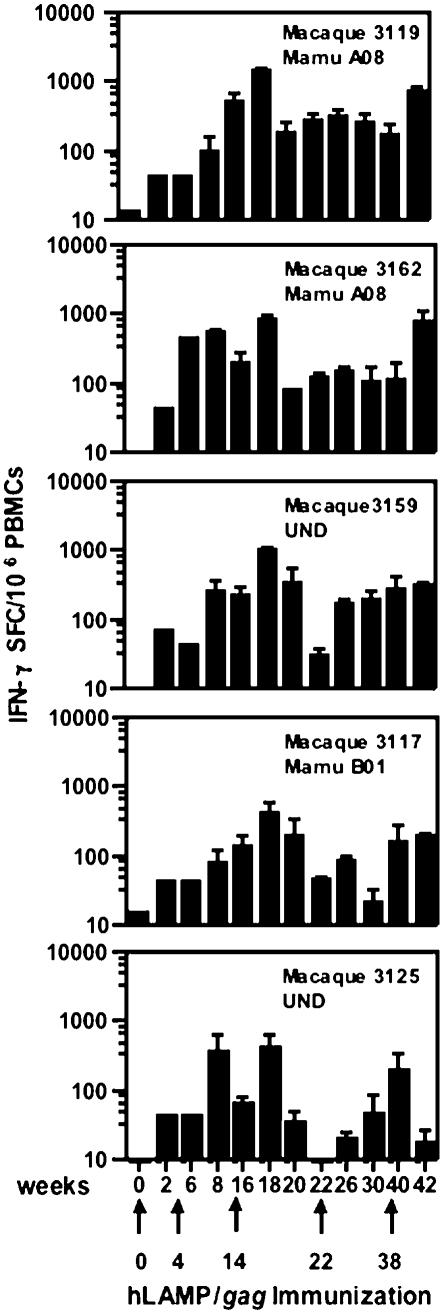
T-cell immune response of immunized macaques. IFN-γ ELISPOT assays were performed with PBMC samples from each of five Rhesus macaques immunized with 5 mg of hLAMP/*gag* DNA plasmid at weeks 0, 4, 14, 22 and 38. Each time point represents the average number of cells activated in separate assays by rGag protein or Gag 15- or 20-aa peptides, performed at two laboratories in duplicate or triplicate. Each bar is the average number of cells activated in 8 to 20 ELISPOT wells+/−S.D. and represents the number of IFN-γ^+^ cells per 10^6^ PBMCs.

**Figure 5:**
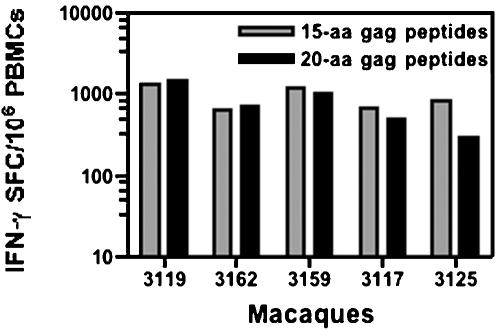
Comparison of the T-cell responses of immunized macaques to Gag 15- and 20-aa peptide pools. Comparison of the average number of IFN-γ^+^ cells activated by Gag 15- and 20-aa peptide pools in the ELISPOT assays, using PBMCs from five individual Rhesus macaques after three DNA immunizations. Each bar represents the number of IFN-γ^+^ cells per 10^6^ PBMCs.

**Figure 6:**
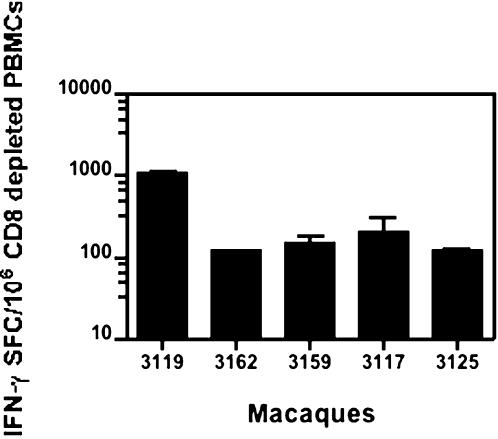
CD4^+^ T-cell immune responses of immunized macaques. ELISPOT assays show the average number of IFN-γ^+^ cells activated by recombinant Gag protein with CD8^+^-depleted PBMCs from five individual rhesus macaques after four DNA immunizations (at week 36). Each bar is the average count from duplicate wells+/−S.D. and represents the number of IFN-γ^+^ cells per 10^6^ CD8^+^-depleted PBMCs.

Five Rhesus macaques were immunized i.m. with 5 mg of the hLAMP/*gag* plasmid at weeks 0, 4, 14, 22 and 38, and the cellular immune responses of each of the individual macaques to the hLAMP/Gag chimera were measured at 12 time points, through 42 weeks (Figure 3). ELISPOT assays to quantify the IFN-γ^+^-secreting cells present in the macaque PBMCs were independently performed at the Southern Research Center and The Johns Hopkins University laboratories. Three different forms of the Gag protein antigen were used to stimulate the PBMCs: HIV rGag and 15- and 20-aa peptide pools that encompass the entire Gag sequence. PBMCs stimulated with all the three formulations of Gag antigen presented similar numbers of IFN-γ^+^ SFCs. These ELISPOT results are presented as the average of the results obtained with the three Gag preparations at the two laboratories. The magnitude of the vaccine-induced T-cell responses varied from macaque to macaque; however, in every case, IFN-γ secretion was detected after the second immunization at week 8, reached the highest level of 600 to 1200 SFC/10^6^ cells at week 18 following the third immunization, and declined gradually until further immunizations were given at weeks 22 and 38 (Figure 4). These studies included an analysis of the relative efficiency of the 15- or 20-aa peptides in stimulating T-cell responses *in vitro*, and we observed that PBMCs of all the five immunized macaques stimulated with the 15- and 20-aa Gag peptide pools separately, showed no significant difference in the IFN-γ response (Figure 5). CD4^+^-specific responses were also studied by ELISPOT analyses with CD8^+^-depleted PBMC stimulated with rGag. All five macaques showed a response in the range of 200–1000 IFN-γ^+^ SFC/10^6^ CD8^+^-depleted cells, indicating a potent CD4^+^-mediated response (Figure 6).

### Mapping of Gag T-cell Immunogenic Determinants

**Figure 7:**
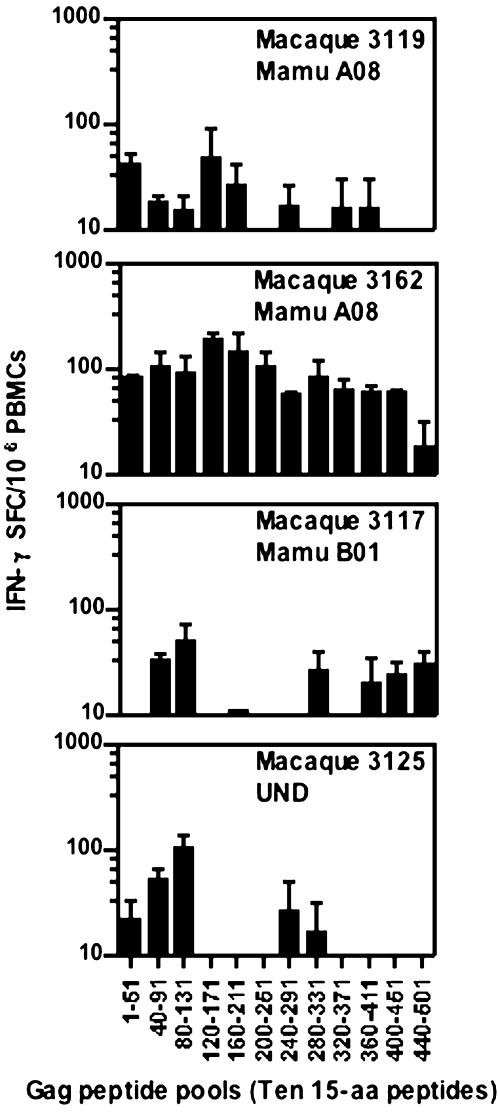
T-cell immune responses to twelve pools of ten 15-aa Gag peptides each. T-cell responses of PBMCs from immunized Rhesus macaques to 12 pools, each consisting of ten gag 15-aa peptides overlapping by 11-aa, were measured by use of ELISPOT assays at week 20 after three DNA immunizations. Each bar represents the frequency of IFN-γ^+^ cells per 10^6^ PBMCs.

The repertoire of peptide-specific responses elicited by lysosomal targeting in BALB/c mice has been shown to include the same immunodominant epitopes as those elicited by the native antigen; however, the chimeric antigen commonly elicits enhanced and additional T-cell responses that were not detected with the native antigen [24]. In an analysis of the potential effect of LAMP targeting on the breadth of the T-cell repertoire in macaques, PBMCs from the immunized macaques were tested at week 20, after 3 DNA immunizations, for T-cell responses to 12 different pools consisting of ten peptides each, of the 15-aa Gag peptide set (Figure 7). T-cell responses to specific peptides were detected with each of the four immunized macaques that were included in this study. Three of the immunized macaques showed responses to five to eight of the peptide pools. One of the animals, macaque #3162, showed activation of IFN-γ^+^ cells in response to all of the peptide pools. Collectively, these data suggest that hLAMP/*gag* can prime a broad T-cell response in primates.

### B-cell Responses of Rhesus Macaques to the hLAMP/*gag* DNA Plasmid

**Figure 8:**
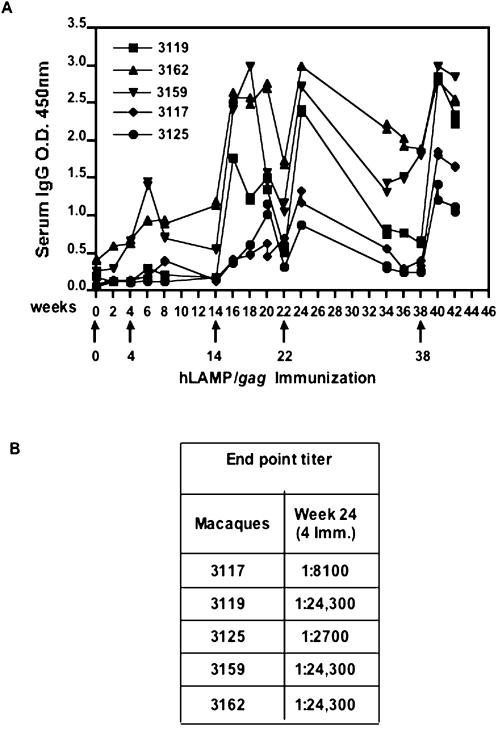
Humoral immune responses of immunized macaques. Gag-specific antibody responses of Rhesus macaques immunized with hLAMP/*gag* plasmid were measured by ELISA. (A) Sera diluted 1∶100 from individual macaques was assayed for total anti-Gag antibodies (IgG). (B) End-point titers were measured at week 24 after four DNA immunizations. The reported titers correspond to the reciprocal of the highest serum dilution that gave an OD value three times higher than that of the corresponding dilution of a non-immune serum.

One remarkable feature of the DNA-encoded LAMP-targeted antigens has been the dramatic increase in antibody-mediated responses of immunized mice. Similar findings have been obtained with the five immunized macaques, each of which showed strong humoral immune responses (Figure 8). The strength of these responses by the individual macaques followed the same pattern as the T-cell responses. High anti-gag IgG antibody titers were present in serum after immunization with hLAMP/*gag*, indicating effective priming. Four of the five vaccinated macaques (#3119, 3162, 3159 and 3117) developed antibodies against HIV-1 after the second immunization and high antibody titers were achieved by these four macaques following the third DNA immunization at week 14. The remaining macaque (#3125), which showed the weakest T-cell response, also elicited a relatively weak antibody response as compared to the other 4 macaques (Figure 8A). The third DNA immunization worked as a booster for all the macaques, and antibody titers reached their highest values after the 5th injection (at week 38). The end-dilution titers of the IgG antibodies achieved after four hLAMP/*gag* immunizations ranged from 2,700 to 24,300 (Figure 8B).

**Figure 9:**
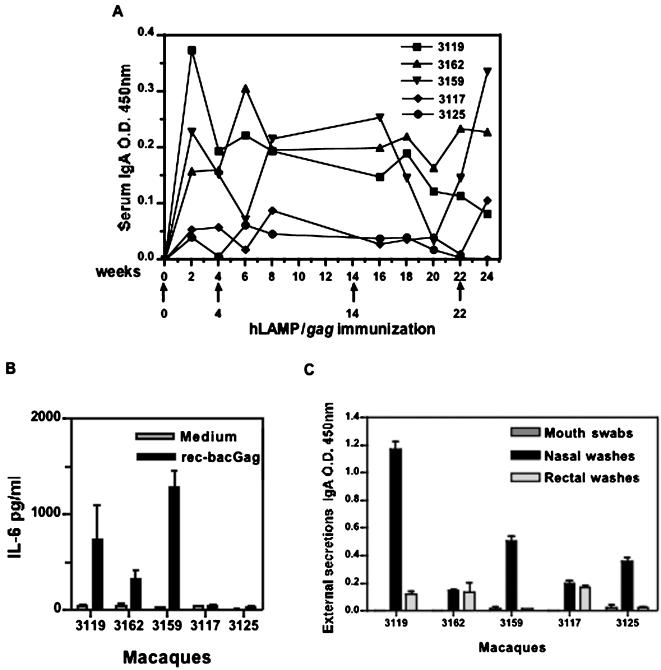
IgA and IL-6 immune responses of hLAMP/*gag* immunized macaques. Gag-specific mucosal immune responses of immunized Rhesus macaques were quantitated by ELISA for IgA and IL-6. (A) Serum IgA levels of the individual macaques at different time-points. (B) IL-6 production of PBMCs from individual macaques stimulated at week 32 with recombinant Gag protein after four DNA immunizations. (C) IgA levels of external secretions of individual macaques.

Serum samples of the immunized macaques were also assayed for IgA production, Significant levels were detected in three (#3119, 3159 and 3162) of the immunized macaques (Figure 9A). IL-6 is a key cytokine for terminal differentiation of B-cells into IgA-secreting plasma cells in both the mouse and human systems [29]–[30]. Interestingly, IL-6 production was seen in the same three macaques (Figure 9B), correlating with the plasma IgA response. The presence of Gag-specific IgA in external secretions, including mouth swabs and nasal and rectal washes collected after four DNA immunizations, was also assayed (Figure 9C). The levels of IgA antibodies detected in mouth swabs and nasal and rectal washes varied remarkably among the individual macaques; however, comparatively higher IgA antibodies were found in nasal washes of all the five macaques.

### B-cell Peptide Epitope Mapping

**Figure 10:**
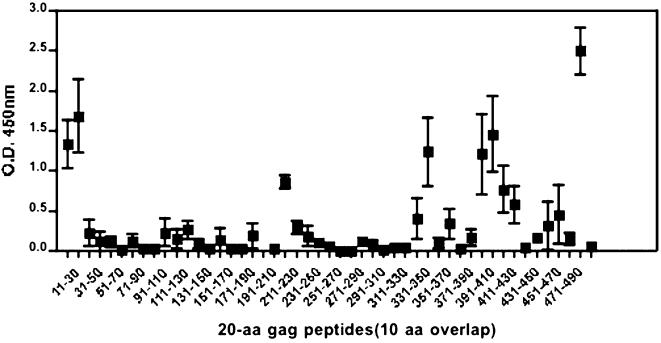
Mapping of B-cell epitopes recognized by immunized macaques. Responses to 49 individual 20-aa Gag peptides overlapping by 10-aa comprising the HIV Gag sequence were measured by ELISA in sera collected at week 24. The results are shown as the average responses of the five macaques. The antibody activity in each of the five macaques was directed at 13 peptides, with dominant responses to 2 amino-terminal and 10 carboxy-terminal sequences.

Serum samples collected from the five macaques at week 24, after four DNA immunizations, were analyzed individually in ELISA assays against each of the 20-aa Gag peptides, overlapping by 10-aa (Figure 10). The repertoires of peptides recognized by each of the macaque sera were very similar; all of the animals reacted with the same 13 of the 49 Gag 20-aa peptides tested. Three peptides containing B-cell determinants were located at the amino-terminal, whereas, most of the peptides recognized were located in the carboxy-terminal region of the Gag protein. These results from immunized macaques support the conclusion that vaccination with an hLAMP/*gag* chimera can prime a broad B-cell response [24].

## Discussion

This study demonstrates that Rhesus macaques immunized with a DNA plasmid vaccine-encoding *gag* as an hLAMP/*gag* chimera develops strong antigen-specific humoral responses as well as CD4^+^ and CD8^+^ T-cell responses. Previous studies by others with candidate DNA vaccines have shown protection of small animals against pathogenic challenges [31]; however, their performance in primates has generally been disappointing. Most naked DNA vaccines have produced sub-optimal immune responses, even with repeated boosting, with only moderate T-cell responses when compared to live-attenuated virus or recombinant virus vaccines and very low or no humoral responses. In contrast, hLAMP/*gag* DNA immunization elicited potent CD4^+ ^T-cell as well as Gag-specific CD8^+ ^T-cell responses in macaques after only a few DNA immunizations. The responses included highly significant IgG antibody titers after two DNA immunizations in four of the five immunized macaques and IgA responses in serum and nasal washes. All of the macaques showed a high IgG antibody titer after three DNA immunizations, with a rapid and enhanced immune memory response with the fourth and fifth immunization.

The breadth of the T and B cell repertoire elicited by DNA HIV vaccines is thought to be important for pathogen control, perhaps by preventing the selection of escape mutants. We have previously reported that immunization of BALB/c mice with LAMP-targeted Gag increases the breadth of B- and T-cell responses. Moreover, others have shown that human dendritic cells transfected with LAMP-targeted HIV nef are able to induce the activation of an increased repertoire of T-cells isolated from infected patients [26]. In this study, each of the four macaques tested, produced Gag-specific IFN-γ^+^ responses to many Gag 15-aa peptide pools, indicating a broad range of T-cell responses. A question that remains to be investigated is whether this result reflects a true increase in the number of new epitopes being recognized as a result of hLAMP/*gag* administration or whether the ability of hLAMP/*gag* to increase the overall magnitude of the cellular immune response simply enhances our ability to detect epitopes that would otherwise have been below the limit of detection.

We also mapped B-cell epitopes of linear, 20-aa Gag peptides with serum samples obtained from the macaques after three DNA immunizations with the hLAMP/*gag* chimera. The results of these analyses indicated that some of the peptides behaved as dominant epitopes and induced strong B-cell activation in all five macaques. These include two dominant peptides located in the amino-terminal portion of the molecule, and 10 peptides located in the carboxy-terminal portion of the Gag protein. The amino-terminal region is known for its antigenic properties [32]–[34] and has been reported to contain both B and T-cell epitopes and to be relatively conserved among European HIV-1 isolates [33], [35]. The carboxy-terminal region is also known to bind antibodies developed during natural HIV-1 infection in humans [36].

Our studies also showed that IgA antibodies were present in the serum and external secretions of the hLAMP/*gag*-immunized macaques. Furthermore, IL-6 production was detected in three of the five immunized macaques. IL-6 is known to play a key role in the terminal differentiation of IgA-committing B cells into IgA-secreting plasma cells in both mice and humans [29]–[30]. In this study, the increased production of IL-6 by Gag-specific CD4^+^ T cells is consistent with the induction of HIV-specific IgA B-cell responses in addition to systemic IgG antibody responses. IgA and IgG antibodies may function as a first line of defense, preventing HIV/SIV adherence to the mucosal surface or interfering with viral replication through secretory IgA [37].

Although T-cell immune responses induced by DNA immunization are generally moderate, previous studies have demonstrated that DNA prime is very important in heterologous prime-boost immunization regimens with viral vectors. The prime-boost formulations have been shown to induce strong cellular immune responses and protection in malaria [38]–[39] and SIVmac [40]–[41] models. Moreover, the DNA prime and viral boost approach has been shown to be important in increasing the breadth of the response repertoire of viral vector vaccines [42], thereby contributing to the overall immunological protection. There is growing consensus that plasmid DNA represents a particularly good prime immunogen. Therefore, considerable work is now being done to explore the use of bimodal vaccine regimens in which the plasmid DNA is used to prime the immune response and a live recombinant vector is used to boost that immunity [43]. These studies collectively suggest that more work is needed before a DNA approach alone or a DNA prime followed by a viral vector boost is able to completely control the pathogenic challenge in these model systems. A recent report has shown consistent and strong CTL responses to Gag in macaques immunized with DNA_CRL 1005-adjuvant Gag plasmids and boosted with rAd5 encoding gag [44]. The studies have established that a significant percentage of humans respond to both the DNA and adenovirus approaches, and more evaluation of further potent DNA vaccines is clearly warranted.

The current study was limited by the small number of animals and the lack of an HIV challenge system to assess the protective efficacy afforded by the HIV hLAMP/*gag* DNA vaccine. A further goal is to test the effect of LAMP targeting on additional relevant antigens of the SIV challenge model, in collaboration with the G. Pavlakis and B. Felber group (Human Retrovirus Section, Basic Research Laboratory, National Cancer Institute, Frederick, MD, USA).
